# Assessing age discrimination in workplaces: psychometric exploration of the Workplace Age Discrimination Scale (WADS-G)

**DOI:** 10.3389/fpsyg.2024.1345923

**Published:** 2024-04-15

**Authors:** Maria Funk, Timo Lorenz

**Affiliations:** Department of Psychology, Medical School Berlin, Berlin, Germany

**Keywords:** ageism, workplace age discrimination, inter-age contact, intersectionality, diversity, scale validation, confirmatory factor analyses, measurement invariance

## Abstract

In contemporary workplaces characterized by diverse age groups working collaboratively, the assessment of age discrimination as an interpersonal phenomenon has gained heightened significance. This study focuses on adapting and scrutinizing the psychometric properties of the German iteration of the Workplace Age Discrimination Scale (WADS-G). Comprehensive Confirmatory Factor Analysis (CFA) results affirm a robust fit for the unidimensional model. Convergent validity is established through correlations between WADS-G scores and related instruments, while discriminant validity is evidenced by its lack of association with extraversion. Noteworthy findings include a positive correlation with turnover intention and negative correlations with job satisfaction, occupational self-efficacy, and organizational affective commitment. Despite its merits, the predictive efficacy of the WADS-G is notably inferior when juxtaposed with the Workplace Incivility Scale. Its explanatory power for turnover intention is constrained when accounting for variables such as job satisfaction, work environment, neuroticism, and core self-evaluation. Although measurement invariance testing across gender groups reveals scalar to strict measurement invariance, the examination across age groups indicates metric invariance. However, Confirmatory Factor Analyses for the 18–30 and 50+ age groups, central to the research emphasis, reveal suboptimal model fit. These outcomes prompt a nuanced discussion on whether the WADS-G aptly captures age-discriminatory experiences across diverse age and gender cohorts among employees.

## 1 Introduction

McClellan and Beggan (2017) conducted qualitative interviews involving both younger and older librarians, analyzing their experiences of negative age-related interactions within a professional context. In their study, they highlighted an illustrative incident: *“When Andrea, 30, gave a faculty member a business card at a networking event, another faculty member commented, “I’ve never seen a graduate student with business cards before—how professional!” […] She felt that this tone captured how her professional activities were diminished when they were associated with her youth.”* Andrea’s experience is not an isolated one (Duncan and Loretto, 2004; Diehl and Dzubinski, 2023), emphasizing the pervasive nature of age-related discrimination. The measurement of such experiences of both younger and older individuals in the workplace, along with the development of strategies for their appropriate management, will become increasingly crucial for practice and research in the years to come.

Demographic changes have played a significant role in shaping the composition of current workplaces. The progressive aging of populations in industrialized societies, along with the increasing labor shortage, represents some of the greatest challenges for organizations. These challenges have led to a notable rise in workforce diversity, marked by the coexistence of different age groups in the workplace (Shultz and Adams, 2019). With a percentage of 22.4 of the population being over 65 years old, Germany ranks seventh among the oldest populations in the world (Richter, 2023). This confronts the country with major challenges. For several years, a gradual increase in the retirement age has been intended to counteract demographic change (Federal Ministry of Labour and Social Affairs, 2022), leading to an increase in labour force participation among individuals aged 60 and above. For the individual, this opens up the possibilities to actively participate in social life for longer and to counteract the threat of poverty in old age (Federal Statistical Office of Germany, 2023). For Organizations, it provides an opportunity to mitigate the labour shortage by either hiring or retaining workers beyond retirement age. From this perspective, this presents a desirable solution to deal with the difficulties of demographic change for both sides.

However, in order to manage these changes constructively and have a lasting positive impact on business success and the people involved, organizations need to look critically at their day-to-day practices and work towards an inclusive workplace (Organization for Economic Cooperation and Development, 2020). To create an inclusive workplace, employees of all ages should feel welcomed and be treated fairly (Rabl and Triana, 2013; Boehm and Kunze, 2015). The reality of such an inclusive culture also involves actively addressing existing negative interpersonal communication patterns and organizational structures.

### 1.1 Workplace age discrimination

Prejudicial, stereotypical and discriminatory interpersonal experiences of older and younger employees and the associated negative consequences have been widely researched (Rudolph and Zacher, 2015; Zacher et al., 2018). A concept that examines these experiences under one umbrella is ageism (Furunes and Mykletun, 2010; Marchiondo et al., 2016; World Health Organization, 2023). Marques et al. (2020) highlighted in their systematic review, that ageism encompasses various facets, incorporating three distinct dimensions: cognitive aspects (i.e., stereotypes), affective aspects (i.e., prejudice), and behavioral facets (i.e., discrimination). This phenomenon operates at both conscious (explicit) and unconscious (implicit) levels and manifests across three tiers: the micro-level (intrapersonal), meso-level (interpersonal and intergroup), and macro-level (institutional and cultural).

Ageism was first defined by Butler (1969, p. 22) as “*a process of systematic stereotyping and discrimination against people because they are old*,” assuming the same theoretical similarities to sexism and racism as the effects of all three “*isms*” are based on the understanding of social class (Butler, 1969) and marginalized group membership. Foundational research work suggests a clear link between prejudiced attitudes toward a marginalized social group and the propensity to discriminate against a target member of that group (Dovidio et al., 1997; Talaska et al., 2008; Jones et al., 2017). Derived from Butler (1969) definition, this means that older people are part of a marginalized group in society and are therefore exposed to discrimination. However, as the topic has been further explored, it has been broadened to also younger individuals and is now characterized by negative attitudes and behaviors towards people solely based on their membership of a particular age group (Greenberg et al., 2002; Levy and Macdonald, 2016). It is furthermore defined by the World Health Organization (WHO) as stereotyping, prejudice, and discrimination towards others regardless of their age groups (World Health Organization, 2023).

In the original study of the Workplace Age Discrimination Scale (WADS), Marchiondo et al. (2016) argued that ageism is dynamic across a person’s lifespan and that the out-groups at greatest risk include older and younger employees, with middle-aged employees forming the in-group [please refer to Ayalon and Tesch-Römer, 2018 for a more in-depth understanding of the theoretical perspectives on ageism]. This is consistent with the findings of their study, as well as the prevailing scientific findings that younger and older employees are more likely to be exposed to negative stereotypes, whilst middle-aged employees are more likely to be judged with positive age-related stereotypes (Duncan and Loretto, 2004; Finkelstein et al., 2015). Despite their evident differences, younger and older employees have equally less influence and fewer resources compared to middle-aged employees (North and Fiske, 2012). Findings even indicate that younger employees are evaluated even more negatively than older employees (Bertolino et al., 2013; Finkelstein et al., 2013). These structures of other-referenced negative stereotypes are reflected in the organizational context by beliefs such as older employees being less competent, less adaptable (Cuddy et al., 2005; Posthuma and Campion, 2009) or slower (Finkelstein et al., 2013) than their middle-aged counterparts. Younger employees are often seen as less conscientious, less emotionally stable or less agreeable (Bertolino et al., 2013). These underlying beliefs have the potential to manifest themselves in social interactions as discriminatory experiences, such as feeling less respected or being treated as less capable.

### 1.2 Measuring workplace ageism

The imperative to accurately gauge attitudes towards older individuals was acknowledged six decades ago, exemplified by the Old People Questionnaire introduced by Tuckman and Lorge (1952). Presently, a plethora of instruments (e.g., Redman and Snape, 2006; Bayl-Smith and Griffin, 2014; Macdonald and Levy, 2016) and methodologies (Fasbender et al., 2023) for assessing age discrimination abound, exhibiting considerable divergence in content, target demographic (young or old), and instrument quality (Ayalon et al., 2019).

A recent systematic review by Peng et al. (2023) on workplace age discrimination measures encompassed evaluations for both younger and older employees, revealing a lack of consensus in the operationalization and assessment of age discrimination. Furthermore, the majority of instruments, originating from the realms of sexism and racism, have not undergone adequate development and validation. An encouraging outlier in this landscape is the WADS by Marchiondo et al. (2016), which underwent an independent development process. Demonstrating commendable psychometric properties, the scale exhibited configural and metric invariance across age groups, with Peng et al.’s (2023) findings even indicating scalar invariance. Nevertheless, the divergent paths taken in the psychometric development and validation within this field present a challenge, precluding meaningful comparisons of results.

In pursuit of enhancing uniformity in the instruments employed, the decision was made not to create a new tool but to adopt and adapt the WADS for German-speaking regions. Aligned with the in-group-out-group perspective articulated by Marchiondo et al. (2016), the WADS focuses on the meso-level of ageism, delving into interpersonal and intergroup-specific experiences of workplace age discrimination (see Table 1). Recent empirical studies have used the scale primarily to better understand age discrimination against older people in the workplace, for example to investigate the consequences of age discrimination and mediating mechanisms (McConatha et al., 2022; Dong et al., 2023; Peng et al., 2023) and antecedents of age discrimination (Reeves et al., 2021; Lagacé et al., 2023; Von Humboldt et al., 2023). But also, to develop new scales (Wilckens et al., 2021) or to validate newly developed scales (Reeves et al., 2021). In consonance with the developmental focus of the WADS and similar research initiatives (Finkelstein et al., 2013), as well as the use of the scale in recent studies a three-part age categorization of employees was instituted for this study: 18–30, 31–49, and 50 + .

**Table 1:** WADS items of the German and English version.

Item	German	English (Marchiondo et al., 2016)
1	Ich wurde aufgrund meines Alters für eine Arbeitsrolle/Aufgabe nicht berücksichtigt	I have been passed over for a work role/task due to my age
2	Meine Beiträge werden aufgrund meines Alters nicht so sehr geschätzt	My contributions are not valued as much due to my age
3	Ich habe aufgrund meines Alters weniger Möglichkeiten erhalten, meine Ideen zu äußern	I have been given fewer opportunities to express my ideas due to my age
4	Ich wurde aufgrund meines Alters unfairerweise weniger wohlwollend bewertet	I have unfairly been evaluated less favorably due to my age
5	Ich erhalte aufgrund meines Alters weniger soziale Unterstützung.	I receive less social support due to my age
6	Ich wurde so behandelt, als ob ich aufgrund meines Alters weniger fähig wäre	I have been treated as though I am less capable due to my age
7	Ich bin aufgrund meines Alters mit weniger Respekt behandelt worden	I have been treated with less respect due to my age
8	Jemand hat meine Anfragen aufgrund meines Alters hinausgezögert oder ignoriert	Someone has delayed or ignored my requests due to my age
9	Jemand hat mich aufgrund meines Alters für Misserfolge oder Probleme verantwortlich gemacht	Someone has blamed me for failures or problems due to my age

English Items are from the original study by Marchiondo et al. (2016).

## 2 Aim of the study

The present study had several objectives. Firstly, it aimed to assess the psychometric properties of the German version of the Workplace Age Discrimination Scale (WADS-G). Secondly, it sought to validate the WADS-G by comparing it with relevant organizational outcome measures. Thirdly, it aimed to establish convergent validity by comparing the WADS-G with measures of perceived age-related mistreatment (Bibby, 2008; BIS) and generic workplace mistreatment (Cortina et al., 2001; Cortina and Magley, 2009; Workplace Incivility Scale; WIS) to ensure their distinctiveness. Fourthly, it sought to ascertain discriminant validity by comparing the WADS-G with extraversion, following the approach used in the study by Marchiondo et al. (2016).

Fifthly, the study aimed to explore the incremental validity of the WADS-G by examining its ability to predict work-related variables beyond age and generic workplace mistreatment. Additionally, it tested whether the WADS-G could predict turnover intentions while controlling for job satisfaction, individual factors (core self-evaluation and neuroticism), and environmental differences (work environment). Lastly, the study aimed to establish measurement invariance across age and gender groups, facilitating comparative studies involving the WADS-G and diverse samples.

To assess the external validity of the WADS-G with relevant organizational outcome measures, the study selected measures similar to those used in the original study (Marchiondo et al., 2016) and the systematic review on workplace age discrimination measures (Peng et al., 2023). These measures included job satisfaction, turnover intention, occupational self-efficacy, and organizational affective commitment. Anticipated correlations included a positive association between the WADS-G and the negative organizational outcome measure (turnover intention) and negative associations with all positive organizational measures (e.g., job satisfaction).

### 2.1 Job satisfaction

Job satisfaction is one of the most extensively studied constructs in organizational research (Judge et al., 2017). Defined as a “*pleasant emotional state arising from the appraisal of one’s work or job experience*” (Locke, 1969, p. 316), this construct involves cognitive, affective, and behavioral reactions (Hulin and Judge, 2003) that are associated with various work-related factors. Job satisfaction has been found to positively impact performance (Judge and Bono, 2001; Harter et al., 2002), reduce turnover intentions (Judge and Klinger, 2008), and correlate with lower absenteeism (Scott and Taylor, 1985). Previous research suggests that perceived age discrimination predicts less job satisfaction (Marchiondo et al., 2019), with correlations ranging from *r* = −0.18 (Macdonald and Levy, 2016) to −0.47 (Peng et al., 2023). Marchiondo et al. (2016) demonstrated that the association is stronger in employees aged 50 and above (*r* = −0.37) compared to those aged between 18 and 30 (*r* = −0.28) using the English Version of the WADS. This suggests that the magnitude of the correlation might vary depending on age group sample. Therefore, a negative small to moderate correlation between the WADS-G and job satisfaction can be expected.

### 2.2 Turnover intention

Employees with turnover intentions often contemplate terminating their employment and express an intention to seek alternative employment opportunities (Mobley et al., 1978), as they have a “*conscious and deliberate willingness to leave the organization*” (Tett and Meyer, 1993, p. 262) and often lack a good identification with work and their organization (Bakker et al., 2004; Qureshi et al., 2013). A high turnover intention rate engenders significant financial implications for companies, manifesting in elevated costs such as productivity losses and heightened error rates among overburdened employees (O’Connell and Kung, 2007). Moreover, it incurs overt costs associated with the actual execution of resignations (Tracey and Hinkin, 2008). Previous research suggests a moderate to strong positive correlation between turnover intention and perceived age discrimination, with correlations ranging from *r* = 0.28 to 0.52 (Marchiondo et al., 2016; Peng et al., 2023). Meta-analytical findings suggest that there are no significant differences in the bivariate correlations between chronological age and turnover intention (Healy et al., 1995). Therefore, a positive moderate to strong correlation between the WADS-G and turnover intention can be expected.

### 2.3 Occupational self-efficacy

General self-efficacy pertains to an individual’s subjective belief in their capability to effectively manage challenging demands through personal actions and abilities (Bandura, 1993). In an occupational setting, self-efficacy refers to the confidence a person feels regarding their ability to successfully fulfill the tasks involved in their job (Bandura, 1977; Schyns and Von Collani, 2002; Rigotti et al., 2008). Those displaying high self-efficacy are distinguished by heightened motivation, a conscious inclination to establish ambitious goals, and the utilization of personal strengths to accomplish their goals (Luthans et al., 2007). In a study conducted by Fasbender and Gerpott (2021), it was found that older employees who perceived age discrimination exhibited lower levels of occupational self-efficacy, as theorized by the authors from a social-cognitive perspective. This perspective pertains to the intertwining of an individual’s self-image with their social identity. In the context of perceived age discrimination, it specifically targets individuals as members of a social group (e.g., colleagues from the same age group), thereby influencing their self-image (Tajfel, 1974; Turner and Reynolds, 2003).

In further examination, this implies that if younger or older employees become aware of their association with a devalued group, this might trigger negative thoughts about how out-group members perceive their social group, leading to a potential impact on their self-assessment of their own skills (Levy, 2003, 2009). Previous research suggests a small to moderate negative correlation between perceived age discrimination and occupational self-efficacy, with correlations ranging from *r* = −0.15 (Furunes and Mykletun, 2010) to −0.32 (Peng et al., 2023). Therefore, a negative small to moderate correlation between the WADS-G and occupational self-efficacy can be expected.

### 2.4 Organizational affective commitment

Organizational commitment is conceptualized as a psychological state that fosters a strong attachment between individuals and their respective organizations (Allen and Meyer, 1990). The subfacet, affective commitment is characterized as “*the employee’s emotional attachment to, identification with, and active involvement in the organization*” (Meyer and Allen, 1991, p. 67). In a study conducted by Rabl and Triana (2013), it was found that perceived age discrimination was associated with lower levels of affective organizational commitment. Drawing from conservation of resources theory, the authors found that this association was even stronger for older employees than for younger employees. In further examination, this implies that older employees appear to be more vulnerable to the stressor of perceived age discrimination and more motivated to conserve resources by reducing their affective organizational commitment than their younger colleagues. In line with these findings, prior research suggests a small to moderate negative correlation between perceived age discrimination and organizational affective commitment, with correlations ranging from *r* = −0.15 (Peng et al., 2023), −0.35 (Redman and Snape, 2006; Furunes and Mykletun, 2010) to *r* = −0.39 (Peng et al., 2023). Therefore, a negative small to moderate correlation between the WADS-G and organizational affective commitment can be expected.

### 2.5 Control variables

It was controlled for neuroticism, core self-evaluations, and workplace age composition. Neuroticism was included to examine whether reports of age discrimination were associated with theoretical outcomes independent of an individual’s disposition to experience negative effects (Gerlitz and Schupp, 2005). Individuals with higher levels of neuroticism might report higher levels of discrimination due to their tendency. Similarly, core self-evaluation (CSE) was integrated based on similar considerations. CSE is considered a stable personality trait and has been linked to job satisfaction in previous research. It represents fundamental appraisals that individuals make about themselves, particularly pertaining to their own worthiness and capabilities (Chang et al., 2012). Individuals with lower levels of core self-evaluation (CSE) might be more prone to reporting higher levels of discrimination, as their existing lower self-perceptions of worthiness and capabilities might make them more susceptible to perceiving instances of discrimination. Workplace age composition was included because of metanalytic findings by Marques et al. (2020), which identified workplace composition as an institutional determinant of ageism.

## 3 Materials and methods

### 3.1 Participants

The sample comprised a total of 673 participants. However, 127 individuals were excluded from the analysis due to incomplete responses, and one participant was excluded because their age did not meet the criteria of inclusion. The final retrained sample consisted of 545 participants *(N_female_ = 273; N_male_ = 270; N_non-binary_ = 1; N_18-30_ = 139; N_31-49_ = 238; N_50+_ = 168)* with an average age of 40.90 years (*SD* = 11.88; Range: 18–66). In this study, 45.61% (*n* = 249) held a university degree, 65.20% (*n* = 356) of the participants were employed full-time and 25.27% (*n* = 138) worked part-time. The remaining 9.36% (*n* = 51) of the sample were either apprentice, working students or civil servants. On average, participants worked 36.20 h per week (*SD* = 16.07) and had 19.66 years of working experience (*SD* = 11.89). The majority of participants (67.77%) stated their current occupation to be in the groups “*health care, social affairs, and education*” (*n* = 148), “*commercial services, retail, sales and distribution, hotels and tourism*” (*n* = 131), “*company organization, accounting, law and administration*” (*n* = 91). The participation was voluntary, and no compensation was provided. The survey was conducted in German. Participants were recruited through personal and professional networks, as well as various social media platforms. Participation requirements included a minimum age of 18 years, a weekly working time of 10 h, and non-self-employment. Participants worked in rather urban environments (*M_work residence_* = 4.40, *SD* = 1.03) and on average, had an age-diverse work environment with younger and older colleagues (*M_work composition_* = 3.01, *SD* = 0.84).

### 3.2 Instruments

#### 3.2.1 Workplace age discrimination scale

The nine items of the English-language original version of the WADS (Marchiondo et al., 2016) were translated into German using the committee-based approach (Brislin, 1980; Furukawa et al., 2014) of the back-translation method (Brislin, 1970). Four individuals, native German speakers with advanced English proficiency, independently translated the items into German before the translation was discussed together, and a consensus was reached on a unified version. Subsequently, the German items were back-translated into English by three bilingual individuals who were fluent in both German and English. Adjustments regarding the wording of the items were made afterwards. The aim was to create German items that accurately conveyed the meaning of the original items while remaining as close as possible to the wording of the English words.

To assess age discrimination at work, participants were asked to rate the nine items on a 5-point rating scale, ranging from 1 = “*Strongly Disagree*” to 5 = “*Strongly Agree*” (e.g.”*My contributions are not valued as much due to my age*”). For the full scale, please see Table 1. With a Cronbach’s α of 0.91 to 0.95 and McDonald’s Omega ω_t_ of 0.93 to 0.97, the WADS-G demonstrates high reliability.

#### 3.2.2 Job satisfaction

Job satisfaction was assessed using three German items (Judge et al., 1994; Judge and Klinger, 2008). The first item measured general job satisfaction (“*All things considered are you satisfied with your job?*”), which participants were able to answer with “*yes*” or “*no*.” The second item (“*How satisfied are you with your job in general?*”) was rated using a 5-point rating scale from 1 = “*very dissatisfied*” to 5 = “*very satisfied*.” The third item asked participants to rate the percentage of time they feel satisfied, unsatisfied or neutral with their job in general (e.g., “*The percent of time I feel satisfied with my present job*.”). The analysis was conducted using the mean-score of the z-standardized items. With a Cronbach’s α of 0.68 to 0.80 and McDonald’s Omega ω_t_ of 0.74 to 80, the scale demonstrates high reliability.

#### 3.2.3 Turnover intention

Intention to leave their current job was assessed with the German Turnover Intention Scale proposed by Böhm (2008). On a five-point rating scale ranging from 1 = “*strongly disagree*” to 5 = “*strongly agree*” participants rate three statements such as “*I often think about leaving my job at my current company.*” With a Cronbach’s α of 0.81 to 0.89 and McDonald’s Omega ω_t_ of 0.82 to 0.89, the scale demonstrates high reliability.

#### 3.2.4 Occupational self-efficacy

Occupational self-efficacy was assessed with the short version of the German Occupational Self-Efficacy Scale (OSS-SF; Rigotti et al., 2008). Six items, such as “*When I am confronted with a problem in my job, I can usually find several solutions.*” are rated on a six-point rating scale (from 1 = “*not at all true*” to 6 = “*completely true*”). With a Cronbach’s α of 0.88 to 0.93 and McDonald’s Omega ω_t_ of 0.92 to 0.94, the scale demonstrates high reliability.

#### 3.2.5 Affective organizational commitment

Affective organizational commitment was assessed with the developed scale by Felfe and Franke (2012), who translated and adapted Meyer and Allen’s (1991) scale to the German context. On a five-point rating scale ranging from 1 = “*do not agree at all*” to 5 = “*do completely agree*” participants rate three statements such as “*I think that my values align with those of the organization.*” With a Cronbach’s α of 0.89 to 92 and McDonald’s Omega ω_t_ of 0.90 to 0.93, the scale demonstrates high reliability.

#### 3.2.6 Perceived generic workplace mistreatment—incivility

Incivility was assessed using the German version of the WIS (Jiménez et al., 2018). Via eight items, participants were asked to rate the frequency of supervisor incivility and coworker incivility, respectively (e.g., “Ignored me or did not respect my opinion”) on a rating scale ranging from 0 = “never” to 6 = “daily.” With a Cronbach’s α of 0.89 to 0.91 and McDonald’s Omega ω_t_ of 0.92 to 0.93, the scale demonstrates high reliability.

#### 3.2.7 Perceived age-related workplace mistreatment—discrimination

Age-related workplace mistreatment was assessed with a German-translated version of the age discrimination scale developed by Bibby (2008). The translation was carried out following the same approach as applied in the case of the WADS-G. Participants were asked to rate four items on a 5-point rating scale, ranging from 1 = “strongly disagree” to 5 = “strongly agree” (e.g., “At work, I sometimes feel that my age is a limitation”). With a Cronbach’s α of 0.81 to 92 and McDonald’s Omega ω_t_ of 0.87 to 93, the scale demonstrates high reliability.

#### 3.2.8 Extraversion

Extraversion was assessed using the German version of the Extraversion dimension of the BFI-S scale (Gerlitz and Schupp, 2005). Participants were asked to rate three items on a 7-point rating scale, ranging from 1 = “*does not apply at all*” to 7 = “*applies completely*” (e.g., “*I am someone who is outgoing, sociable*”). With a Cronbach’s α of 0.95 to 96 and McDonald’s Omega ω_t_ of 0.95 to 0.96, the scale demonstrates high reliability.

#### 3.2.9 Neuroticism

Neuroticism was assessed using the German version of the Neuroticism dimension of the BFI-S scale (Gerlitz and Schupp, 2005). Participants were asked to rate three items on a 7-point rating scale, ranging from 1 = “*does not apply at all*” to 7 = “*applies completely*” (e.g., “*I am someone who often worries*”). With a Cronbach’s α of 0.91 to 0.95 and McDonald’s Omega ω_t_ of 0.91 to 0.95, the scale demonstrates high reliability.

#### 3.2.10 Core self-evaluations

Core self-evaluations was assessed with the German version of the Core Self-Evaluation Scale (G-CSES; Heilmann and Jonas, 2010). The G-CSES consists of 12 statements (“*I am confident I get the success I deserve in my life*”). Participants rated these items on a five-point rating scale from 1 = “*strongly disagree*” to 5 = “*strongly agree*.” With a Cronbach’s α of 0.91 to 0.94 and McDonald’s Omega ω_t_ of 0.94 to 0.95, the scale demonstrates high reliability.

#### 3.2.11 Demographics

Participants were asked to state their age, gender, highest level of completed education, employment status, weekly working hours, how long they have been working and sector of employment encoded with the classification of occupations 2010 (Bundesagentur für Arbeit, 2011). Further, the participants were asked about the approximate ratio of younger and older people in their workplace (workplace composition; ranging from 1 = “predominantly older people” to 5 = “predominantly younger people”).

### 3.3 Data analysis

To test the fit of the measurement models, the criteria proposed by Hu and Bentler (1999) were used. Beyond χ^2^ significance testing, these criteria comprise a standardized root-mean-square residual (SRMR) ≤ 0.08 in combination with at least one of the following fit indices: a root-mean-square error of approximation (RMSEA) ≤ 0.06, a lower bound of the 90% confidence interval of the RMSEA ≤0.06, a comparative fit index (CFI) ≥ 0.95, or a Tucker Lewis index (TLI) ≥ 0.95. The confirmatory factor analyses were conducted using the package “*Lavaan*” (Rosseel, 2012), as well as the packages “*Hmisc*” (Harrell, 2022), “*psych*” (Revelle, 2021) und “*semPlot*” (Epskamp, 2019) with the software R (R Core Team, 2014).

To assess internal consistency, in addition to Cronbach’s α, McDonald’s ω_t_ was employed (McDonald, 1999). To evaluate divergent and convergent validity, Pearson’s correlation coefficients were calculated with other relevant measures. Correlations were evaluated as follows: correlations >0.1–small, >0.3–moderate, and > 0.5–strong.

To assess incremental and construct validity, hierarchical regression analysis was performed. However, due to the high correlation between the workplace incivility scale (WIS), the other workplace age discrimination scale (BIS), and the WADS-G, the assumption of multicollinearity is violated (Graham, 2003). To address this, a relative weight analysis (RWA; Lebreton et al., 2007; Tonidandel and LeBreton, 2010) was subsequently conducted. This approach helps in gaining a better understanding of the relative importance of each predictor. RWA Web (Tonidandel and LeBreton, 2015) was used, specifying bootstrapping with 10,000 replications and a 0.05 alpha level.

For measurement invariance testing with a small sample size (total *N* < 300), unequal sample sizes and mixed lack of invariance, the following cut-offs proposed by Chen (2007) were applied. For testing loading invariance, a change of ≤ − 0.05 in the CFI, in addition with a change of ≥0.010 in RMSEA, or a change of ≥0.025 in SRMR indicates non-invariance. For testing intercept or residual invariance, a change of ≥ −0.005 in CFI, in addition with a change of ≥0.010 in RMSEA or a change of ≥0.005 in SRMR indicates non-invariance.

## 4 Results

### 4.1 Structural validity

#### 4.1.1 Psychometric properties of the WADS-G

**Figure 1:**
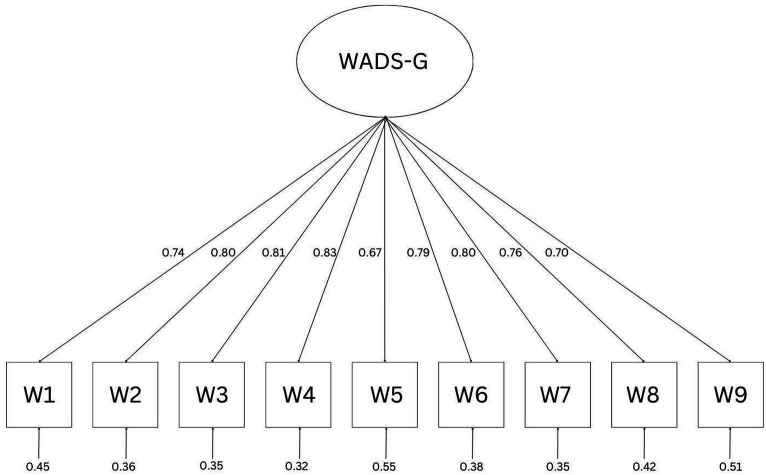
Measurement model of the WADS-G with standardized loadings.

Based on the theoretical considerations by Marchiondo et al. (2016), it was examined whether the WADS-G represents a unidimensional construct. Considering the criteria for fit indices (Hu and Bentler, 1999), the WADS-G showed a good model fit with Satorra-Bentler-*χ*^2^ (27, *N* = 545) = 37.246, *p* = 0.091, CFI = 0.979, TLI = 0.971, RMSEA = 0.065, 90%- CIRMSEA [0.000, 0.111], SRMR = 0.036 (Table 2). The standardized loadings ranged from 0.67 to 0.83 and can be fully viewed in Figure 1.

**Table 2:** Confirmatory factor analyses for the used scales.

CFI	TLI	RMSEA	RMSEA [CI]	SRMR
0.979	0.971	0.065	0.000–0.111	0.036
0.961	0.945	0.059	0.089–0.063	0.035
0.971	0.913	0.161	0.085–0.250	0.033
0.991	0.985	0.059	0.000–0.113	0.022
1.000	1.004	0.000	0.000–0.050	0.007
0.849	0.816	0.145	0.131–0.160	0.065

*N* = 545. JS, TI, EX and NE have only three items each which creates a CFA model with zero degrees of freedom and is therefore not testable; WADS-G, German version of the workplace age discrimination scale; WIS, workplace incivility scale; BIS, Bibby’s workplace age discrimination scale; OSE, occupational self-efficacy scale; AC, organizational affective commitment scale; CSE, core self-evaluation scale; the scales for jobs satisfaction, turnover intention, extraversion and neuroticism have only three items each which creates a CFA model with zero degrees of freedom and is therefore not testable.

#### 4.1.2 Psychometric properties of the other used scales

Considering the criteria for fit indices (Hu and Bentler, 1999), the occupational self-efficacy scale (OSE) and the organizational affective commitment scale (AC) showed a good model fit. The workplace incivility scale (WIS) and Bibby’s age discrimination scale (BIS) showed a moderate model fit while the core self-evaluation scale (CSE) showed an insufficient model fit. Psychometric properties of all scales with more than three items are provided in Table 2.

#### 4.1.3 Descriptive analyses and external validity

Most of the scales utilized in the analysis demonstrate non-normal distributions, as indicated by the results of significant Shapiro–Wilk tests (*p* < 0.001). Descriptive statistics, McDonald’s *ω*_t_, Cronbach’s α and bivariate correlations with confidence intervals are presented in Table 3. The data provides support for all proposed postulates and indicates evidence for the external validity of the scale. The WADS-G exhibited a small negative correlation with job satisfaction (*r* = −0.21), a moderate negative correlation with occupational self-efficacy (*r* = −0.37) and affective organizational commitment (*r* = −0.33), as well as a moderate positive correlation with turnover intention (*r* = 0.35, all at *p* < 0.001).

**Table 3:** Descriptives and bivariate correlations with confidence intervals for the overall sample.

Variable	M	SD	1	2	3	4	5	5	7	8	9	10	11
1. Age	40.90	11.80											
2. WADS	1.18	0.45	−0.02	0.94 (0.92)									
			[−0.11, −0.06]										
3. JS	1.69	0.49	0.05	−0.21***	0.77 (0.76)								
			[−0.04, 0.13]	[−0.29, −0.13]									
4. TI	1.92	1.10	−0.26***	0.35***	−0.47***	0.87 (0.87)							
			[−0.33, −0.18]	[0.28, 0.42]	[−0.53, −0.40]								
5. OSE	5.10	0.76	−0.09*	−0.37***	0.36***	−0.45***	0.92 (0.92)						
			[0.01, 0.18]	[−0.44, −0.29]	[0.28, 0.43]	[−0.51, −0.38]							
6. AC	3.92	0.97	0.11*	−0.33***	0.54***	−0.78***	0.50***	0.91 (0.91)					
			[0.02, 0.19]	[−0.40, −0.25]	[0.48, 0.60]	[−0.81, −0.75]	[0.43, 0.56]						
7. WIS	1.58	0.68	−0.04	0.57***	−0.35***	0.46***	−0.44***	−0.51**	0.93 (0.90)				
			[−0.12, 0.04]	[0.51, 0.62]	[−0.42, −0.27]	[0.39, 0.52]	[−0.51, −0.37]	[−0.57, −0.45]					
8. BI	1.22	0.48	0.09*	0.74***	−0.19***	0.24***	−0.27***	−0.26**	0.50***	0.89 (0.85)			
			[0.01, 0.18]	[0.70, 0.78]	[−0.27, −0.11]	[0.16, 0.32]	[−0.35, −0.19]	[−0.33, −0.18]	[0.44, 0.56]				
9. EX	4.57	2.02	−0.00	0.06	0.10*	0.12**	−0.04	−0.08	0.05	0.06	0.96 (0.96)		
			[−0.09, 0.08]	[−0.03, 0.14]	[0.02, 0.18]	[0.03, 0.20]	[−0.12, 0.05]	[−0.16, 0.00]	[−0.03, 0.14]	[−0.02, 0.15]			
10. NE	3.26	1.84	−0.02	0.10*	−0.17***	0.08	−0.22***	−0.06	0.11**	0.12**	−0.17***	0.93 (0.93)	
			[−0.10, 0.06]	[0.02, 0.18]	[−0.25, −0.09]	[−0.01, 0.16]	[−0.30, −0.13]	[−0.14, 0.03]	[0.03, 0.20]	[0.03, 0.20]	[−0.25, −0.09]		
11. CSE	4.87	0.64	−0.04	−0.54***	0.34***	−0.45***	0.61***	0.52**	−0.59***	−0.44***	−0.09*	−0.23***	0.95 (0.93)
			[−0.13, 0.04]	[−0.59, −0.47]	[0.27, 0.41]	[−0.51, −0.38]	[0.56, 0.66]	[0.45, 0.58]	[−0.64, −0.53]	[−0.51, −0.37]	[−0.17, −0.00]	[−0.31, −0.15]	

*N* = 545; *N*_18-30_ = 139; *N*_31-49_ = 238; *N*_50+_ = 168. M and SD are used to represent mean and standard deviation, respectively. McDonald’s omega (Cronbach’s alpha) is displayed in diagonals if applicable. Values in square brackets indicate the 95% confidence interval for each correlation. The confidence interval is a plausible range of population correlations that could have caused the sample correlation (Cumming, 2014). * indicates *p* < 0.05; ** indicates *p* < 0.01; *** indicates *p* < 0.001.

Furthermore, the WADS-G exhibited a strong positive correlation with measures of age (BIS; *r* = 0.74) and generic workplace mistreatment (WIS; *r* = 0.57, all at *p* < 0.001), still ensuring their distinctiveness and indicating convergent validity of the WADS-G. Also, no significant correlation with extraversion was found, indicating discriminant validity. Additionally, as expected, the WADS-G exhibited a small positive correlation with neuroticism (*r* = 0.10, *p* < 0.05) and a strong negative correlation with core self-evaluation (*r* = −0.54, *p* < 0.001).

#### 4.1.4 Incremental validity

The WIS (*β* = −0.33, *p* < 0.01) was found to be significant negative related to job satisfaction, while the BIS (*β* = −0.02, *p* > 0.05) did not show a significant association. Moreover, the WADS-G did not incrementally predict job satisfaction beyond the other two constructs (*β* = −0.01, *p* > 0.05, △R^2^ = 0.00). The WIS (*β* = 0.45, *p* < 0.01) was found to be significant positive related to turnover intention, whereas the BIS (*β* = 0.01, *p* > 0.05) did not show a significant association. Furthermore, the WADS-G did incrementally predict turnover intention beyond the other constructs (*β* = 0.19, *p* < 0.01, △R^2^ = 0.01). The WIS (*β* = −0.39, *p* < 0.01) was found to be significant negative related to occupational self-efficacy, whereas the BIS (*β* = −0.06, *p* > 0.05) did not show a significant association.

Additionally, the WADS-G did incrementally predict occupational self-efficacy beyond the other constructs (*β* = −0.20, *p* < 0.01, △R^2^ = 0.02). The WIS (*β* = −0.49, *p* < 0.01) was also found to be significant negative related to affective organizational commitment, while the BIS (*β* = 0.05, *p* > 0.05) did not show a significant association. Moreover, the WADS-G did not incrementally predict affective organizational commitment beyond the other constructs (*β* = −0.08, *p* > 0.05, △R^2^ = 0.00). All hierarchical regression analysis and their corresponding details are presented in Table 4.

**Table 4:** Hierarchical regression analyses on inappropriate workplace behavior.

	Model 1		Model 2		Model 3	
	Step 1	Step 2	Step 1	Step 2	Step 1	Step 2
*Job satisfaction*						
WIS	−0.35^***^	−0.33^***^			−0.34^***^	−0.33^***^
BIS			−0.19^***^	−0.08	−0.02	−0.02
WADS-G		−0.03		−0.15^*^		−0.01
R^2^	0.12^***^	0.12	0.03	0.04	0.11	0.12
△R^2^		0.00		0.01		0.00
F	74.98	37.42	20.38	13.54	37.58	24.94
*Turnover intention*						
WIS	0.45^***^	0.39^***^			0.45^***^	0.40^***^
BIS			0.24^***^	−0.05	0.00	0.1
WADS-G		0.12^*^		0.39^***^		0.19^**^
R^2^	0.21^***^	0.21^***^	0.05^***^	0.12^***^	0.21^***^	0.22^***^
△R^2^		0.09		0.07		0.01
F	142.4	74.64	32.52	38.71	71.07	51.29
*Occupational self-efficacy*						
WIS	−0.44^***^	−0.35^***^			−0.41^***^	−0.36^***^
BIS			−0.27	0.00	−0.06	0.06
WADS-G		−0.16^***^		−0.37^***^		−0.20^***^
R^2^	0.19^***^	0.21^***^	0.07^***^	0.13^***^	0.20^***^	0.21^***^
△R^2^		0.02		0.06		0.02
F	132.2	73.11	42.69	41.96	67.09	49.17
*Affective organizational commitment*						
WIS	−0.51^***^	−0.49^***^			−0.51^***^	−0.49^***^
BIS			−0.25^***^	−0.04	0.00	0.05
WADS-G		−0.04		−0.30^***^		−0.08
R^2^	0.26^***^	0.26^***^	0.06^***^	0.10^***^	0.26^***^	0.26^***^
△R^2^		0.00		0.04		0.00
F	192.1	96.15	38.59	32.21	95.87	64.31

*N* = 539; standardized *β*-coefficients; * *p* < 0.05, ** *p* < 0.01, *** *p* < 0.001; WADS-G, German version of the workplace age discrimination scale; WIS, workplace incivility scale; BIS, Bibby’s workplace age discrimination scale.

To better understand the relative importance of each predictor, a relative weight analysis was supplemented. The results show that the three predictors (WIS, BIS, WADS-G) explained 11.10% of the variance in job satisfaction. Each predictor explained significant variance, but their relative weights did differ from one another. Expressed in terms of rescaled relative weights, the WIS explains 77.33% of all variance in job satisfaction explained by the predictors, while the WADS-G has a rescaled relative weight of 11.85% and the BIS of 10.82%, respectively. Accordingly, the results support the finding from the hierarchical regression analyses. In the analysis of the other criterion variables, the results also show that the WADS-G significantly relates to those variables and explains meaningful variance. However, its predictive power is considerably weaker compared to the WIS. Detailed information regarding the weight analyses is presented in Table 5.

**Table 5:** Summary of relative weight analyses.

	Raw RW	Raw [CI]	Sig. [CI]	Res RW in %	R^2^ in %
*Job satisfaction*					
WIS	0.09	[0.048–0.131]	[0.048–0.134]	77.33	11.10
BIS	0.01	[0.005–0.024]	[0.003–0.027]	10.82	
WADS-G	0.01	[0.001–0.026]	[0.003–0.030]	11.85	
*Turnover intention*					
WIS	0.15	[0.091–0.206]	[0.091–0.211]	64.05	22.77
BIS	0.02	[0.001–0.035]	[0.007–0.038]	8.81	
WADS-G	0.06	[0.031–0.104]	[0.029–0.105]	27.14	
*Occupational self-efficacy*					
WIS	0.13	[0.074–0.197]	[0.075–0.202]	59.45	21.84
BIS	0.02	[0.011–0.047]	[0.009–0.050]	11.43	
WADS-G	0.06	[0.034–0.106]	[0.034–0.107]	29.22	
*Affective organizational commitment*					
WIS	0.20	[0.143–0.262]	[0.141–0.265]	74.79	26.51
BIS	0.02	[0.011–0.040]	[0.010–0.043]	8.63	
WADS-G	0.04	[0.024–0.070]	[0.024–0.076]	16.58	

Raw RW, raw relative weight; Raw [CI], raw relative weight lower bound of confidence interval used to test the statistical significance of raw weight und upper bound of confidence interval used to test the statistical significance of raw weight; Sig. [CI], Confidence Interval Tests of significance; Res RW, relative weight rescaled as a percentage of predicted variance in the criterion variable attributed to each predictor; R^2^, total variance explained by the predictors; WADS-G, German version of the workplace age discrimination scale; WIS, workplace incivility scale; BIS, Bibby’s workplace age discrimination scale.

The hypothesized model of job satisfaction, workplace age discrimination, the work environment, neuroticism and core self-evaluation as predictors of turnover intention was tested using hierarchical regression analysis. All hierarchical regression analyses and their corresponding details are presented in Table 6. The results are as expected, as job satisfaction (*β* = −0.36, *p* < 0.001) and workplace age discrimination (*β* = 0.13, *p* < 0.01) are statistically significant predictors of turnover intentions as well as core-self-evaluation (*β* = −0.26, *p* < 0.001). Beyond those constructs, the WADS-G incrementally predicts turnover intention with △R^2^ = 0.01. While work environment (*β* = −0.01, *p* > 0.05) and neuroticism (*β* = −0.06, *p* > 0.05) are not statistically significant predictors of turnover intention.

**Table 6:** Hierarchical regression analyses on turnover intention.

	Model 1		Model 2		Model 3		Model 4		Model 5	
	Step 1	Step 2	Step 1	Step 2	Step 1	Step 2	Step 1	Step 2	Step 1	Step 2
JS^a^	−0.46^***^	−0.41^***^							−0.36^***^	−0.36^***^
WE			−0.04	−0.05					−0.01	−0.01
NE					0.08	0.04			−0.06	−0.06
CSE							−0.44^***^	−0.36^***^	−0.33^***^	−0.26^***^
WADS-G		0.25^***^		0.35^***^		0.35^***^		0.16^***^		0.13^**^
R2	0.22^***^	0.27^***^	−0.00	0.12^***^	0.00	0.12^***^	0.20^***^	0.22^***^	0.30^***^	0.32^***^
△R^2^		0.06		0.12		0.12		0.02		0.01
F	151.3	102.9	0.75	38.88	3.16	38.87	136.7	75.67	60.68	50.82

*N* = 539; standardized *β*-coefficients, * *p* < 0.05, ** *p* < 0.01, *** *p* < 0.001; JS, job satisfaction scale; WE, workplace age composition; NE, neuroticism; CSE, core self-evaluation scale; WADS-G, German version of the workplace age discrimination scale; a, Standardized z-scores.

#### 4.1.5 Measurement invariance

##### 4.1.5.1 Age-groups

Indices of the model for the 18–30 sample revealed an insufficient fit of the unidimensional construct (CFI = 0.86; TLI = 0.82; RMSEA = 0.15; 90% CI RMSEA = (0.08, 0.22), SRMR = 0.07), with standardized loadings ranging from 0.60 to 0.81. Similarly, the model for the age-group +50 sample also demonstrated an insufficient fit (CFI = 0.86; TLI = 0.82; RMSEA = 0.16; 90% CI RMSEA = (0.10, 0.22), SRMR = 0.07), with standardized loadings ranging from 0.59 to 0.93. Among the three age-group samples, only the model in the age group 31–49 demonstrated a moderate fit (CFI = 0.98; TLI = 0.97; RMSEA = 0.08; 90% CI RMSEA = (0.00, 0.17), SRMR = 0.07), with standardized loadings ranging from 0.69 to 0.90.

Measurement Invariance testing between the age-groups revealed a good fit for the configural and metric model (Hu and Bentler, 1999; Table 7). However, according to the chi-square significance test (*p* > 0.30) and the fit indices proposed by Chen (2007), the data shows also metric measurement invariance. Further results showed a lack in scalar invariance, as the chi-square significance test was found to be significant (*p* < 0.001) and the fit indices are not changing within the range that was proposed by Chen (2007). Detailed model fit indices for the age-group samples are presented in Table 7.

**Table 7:** Confirmatory factor analyses and measurement invariance results for gender and age groups.

	*X*^2^ (df)	Sig.	CFI	TLI	RMSEA	RMSEA [CI]	SRMR	△CFI	△RMSEA	△SRMR
**Gender**										
Men	29.121 (27)	0.355	0.992	0.990	0.039	[0.000–0.119]	0.036			
Women	33.442 (27)	0.183	0.972	0.963	0.069	[0.000–0.137]	0.050			
Configural	62.621 (54)	0.197	0.983	0.977	0.056	[0.000–0.110]	0.043			
Metric	65.556 (62)	0.355	0.993	0.991	0.035	[0.000–0.095]	0.069	0.010	−0.022	0.026
Scalar	73.455 (70)	0.366	0.994	0.993	0.030	[0.000–0.087]	0.070	0.001	−0.004	0.001
Strict	90.092 (79)	0.185	0.976	0.978	0.056	[0.000–0.104]	0.085	−0.018	0.025	0.015
**Age**										
18–30	49.729 (27)	0.005	0.864	0.819	0.151	[0.082–0.216]	0.069			
31–49	32.053 (27)	0.230	0.977	0.969	0.077	[0.000–0.165]	0.048			
+50	54.610 (27)	0.001	0.864	0.818	0.156	[0.095–0.216]	0.066			
Configural	127.048 (81)	0.001	0.924	0.899	0.126	[0.082–0.167]	0.059			
Metric	141.922 (97)	0.002	0.917	0.908	0.120	[0.074–0.161]	0.125	−0.006	−0.006	0.066
Scalar	175.575 (113)	<0.001	0.901	0.905	0.122	[0.085–0.156]	0.130	−0.017	0.002	0.005
Strict	224.417 (131)	<0.001	0.805	0.839	0.159	[0.123–0.194]	0.176	−0.096	0.037	0.046

*X*^2^ refers to the Chi-square difference value with respective degrees of freedom (df). Sig. is used to display the *p*-value of the Chi-square difference test. The confirmatory fit index is reported as CFI, the Tucker-Lewis index as TLI. RMSEA is the robust Root Mean Square Error of Approximation with respective 90%-confidence intervals [CI]. The SRMR is the Root Mean Square Residual. CFA means confirmatory factor analysis. MGCFA means multigroup confirmatory factor analysis. △ refers to the change in the indices.

##### 4.1.5.2 Gender-groups

Indices of the model for the men sample revealed a good fit of the unidimensional construct (CFI = 0.992; TLI = 0.990; RMSEA = 0.039; 90% CI RMSEA = (0.000, 0.119), SRMR = 0.036), with standardized loadings ranging from 0.75 to 0.87. Similarly, the model for the women sample also demonstrated a good model fit (CFI = 0.972 TLI = 0.963; RMSEA = 0.069; 90% CI RMSEA = (0.000, 0.137), SRMR = 0.050), with standardized loadings ranging from 0.58 to 0.84.

Measurement Invariance testing between the gender-groups revealed a good fit for the configural, metric, scalar and strict model (Hu and Bentler, 1999; Table 7). Further, the chi-square significance test indicates a strict measurement invariance (*p* > 0.17). However, according to the fit indices proposed by Chen (2007), the data shows no strict measurement invariance. Detailed model fit indices for the gender-group samples are presented in Table 7.

## 5 Discussion

In this study, the WADS-G, an instrument designed to assess workplace age discrimination in the German-speaking population, was translated and validated. WADS-G demonstrated robust psychometric properties and exhibited both convergent and discriminant validity. Furthermore, the WADS-G significantly relates to job satisfaction, turnover intention, occupational self-efficacy as well as affective commitment and explains meaningful variance. However, despite its strengths, the predictive power of the WADS-G is considerably weaker compared to the workplace incivility scale and has limited explanatory power for turnover intention when controlling for job satisfaction, work environment, neuroticism, and core self-evaluation.

The analyses of measurement invariance revealed that age-specific groups demonstrated metric measurement invariance, and gender-specific groups exhibited strict measurement invariance. However, the data suggested that the WADS-G is most effective in capturing age discrimination among employees aged between 31 and 49, while its suitability for the other two age groups is limited. These findings merit critical consideration, particularly since the 18–30 and 50+ groups constitute the central focus groups within the theoretical framework of age discrimination in this research emphasis. Moreover, given the weak predictive power beyond the workplace incivility scale, there arises a question regarding the questionnaire’s ability to comprehensively capture and measure the actual phenomenon in its substantive richness.

### 5.1 Factorial structure

#### 5.1.1 Age group differences

The findings regarding measurement invariance across age groups are consistent with the original study (Marchiondo et al., 2016), indicated that the English version of the WADS showed both configural and metric invariance across age groups. However, when it comes to determining whether the WADS-G effectively measures age discrimination among both younger and older workers, it’s essential to consider the challenge of achieving configural invariance. Configural invariance implies that participants from different groups perceive and understand the underlying constructs in the same way (Riordan and Vandenberg, 1994; Cheung and Rensvold, 2002). Yet, in the context of researching age discrimination, achieving this uniformity in understanding becomes questionable.

This uncertainty arises due to reported variations in experiences of discrimination among younger and older employees in the literature (Bertolino et al., 2013; Finkelstein et al., 2013). Building upon that argument it is worth considering the study results by McClellan and Beggan (2017). Their findings indicate an age-related distinction between two forms of stigma: enacted stigma, characterized by explicit comments about a participant’s age, and felt stigma, where the participant experiences discomfort and self-consciousness related to age. Interestingly, while both older and younger workers report instances of felt stigma, only younger workers report instances of enacted stigma. The authors of the study interpreted their results by suggesting that politeness norms might inhibit people from making direct comments about age, as being older is perceived as a negative characteristic.

This aligns with the perspectives of DeVos and Banaji (2005), who argued that the shift towards increased egalitarian societal values and concomitant legislative reforms has led to a more conscious expression of egalitarian beliefs. This differentiation is reflected in the distinction between overt forms of discrimination, which involve explicit manifestations of discrimination usually observed in the formal aspects of one’s job, and covert forms of discrimination, which entail subtle manifestations occurring in the informal, interpersonal aspects of one’s job (Jones et al., 2014, 2016, 2017). Considering that the Workplace Age Discrimination Scale is primarily designed to assess covert forms of workplace age discrimination (Marchiondo et al., 2016; Peng et al., 2023), there is a high likelihood that distinguishing between uncivil and discriminatory behavior is challenging with the items of the WADS-G. This circumstance potentially leads to metric measurement invariance, yet it underscores that the scale inadequately captures the phenomenon of age discrimination within the theoretical focus groups of younger and older workers.

Furthermore, as emphasized in Ayalon et al.’s (2019) systematic review of existing ageism scales, it is imperative to develop and validate a scale that considers the multidimensional nature of ageism in order to accurately capture a potential increase in the magnitude of this phenomenon. Taken together, this indicates the importance of further exploring the multidimensionality at the level of covert forms of discrimination.

A theoretical approach to address this could involve adopting the two facets of the microaggression taxonomy (Sue, 2010), emphasizing microinsults and microinvalidations. This framework provides a multidimensional perspective on covert workplace age discrimination, capturing the largely imperceptible psychological factor of age stereotypes (Schloegel et al., 2018) while allowing for the assessment of interpersonal communications and instances of enacted stigma (McClellan and Beggan, 2017). Developing a new workplace age discrimination scale should acknowledge the divergent experiences of younger and older workers, necessitating distinct item content for accurate assessment. Algner and Lorenz’s (2022) methodological approach to scale development for gender microaggressions, involving a genetic algorithm, offers possible guidance for future scale development efforts in the field of age discrimination in the workplace.

#### 5.1.2 Gender group differences

The results demonstrated robust psychometric properties for the WADS-G in both gender groups, including strict measurement invariance. However, these findings necessitate discussion in light of the limited predictive power beyond workplace incivility and the scale’s insufficient capture of age discrimination in the focus groups, prompting questions about whether the WADS-G accurately measures the intended construct. The findings on measurement invariance indicate that participants from the two different gender groups share a uniform understanding of the constructs, these findings are not consistent with the current research on gender-age intersectionality in the workplace (Duncan and Loretto, 2004; Purdie-Vaughns and Eibach, 2008; Francioli and North, 2021; Walker and Zelin, 2021).

Gender-age intersectionality in the workplace, often referred to as gendered ageism (Itzin and Phillipson, 1995; Handy and Davy, 2007; Wallenberg and Jansson, 2021; Liddy, 2023), suggests that female employees of all ages are more exposed to age discrimination than their male colleagues, often based on appearance and sexuality (Duncan and Loretto, 2004; Granleese and Sayer, 2006; Clarke and Griffin, 2008). Duncan and Loretto’s (2004) study titled “Never the Right Age?” further indicates that, akin to younger employees, negative attitudes disproportionately affect women due to multiple discrimination. Over the years, various definitions and ongoing discussions have emerged regarding the interplay between age and gender. At a broader level, it can be asserted that the concept recognizes the interconnected nature of individuals’ experiences and identities, influenced by the overlapping effects of both gender and age (Krekula et al., 2018). In their review, Krekula et al. (2018) highlight that while the combined impact of gender and age is frequently illustrated in studies concerning women, there remains a lack of substantial data and comprehensive theoretical exploration regarding the potential and manner in which such concurrent factors influence men.

### 5.2 External validity

The correlations across the group analyses largely align with the theoretical considerations and might provide an indication for the external validity of the WADS-G. However, a noteworthy deviation was observed in the 18–30 sample, where the correlation with occupational self- efficacy was stronger than expected. This might be because younger employees in this sample have the strongest association of age discrimination with specific experiences, such as being given fewer opportunities, treated with less respect or contributions being not valued much due to their age. Considering that individuals with high occupational self-efficacy are known for their heightened motivation, conscious pursuit of ambitious goals, and effective utilization of personal strengths to achieve their objectives (Luthans et al., 2007), it is not surprising that their experiences of workplace age discrimination would strongly impact their occupational self-efficacy. Additionally, in the correlations of the 18–30 sample, the results suggest a moderate positive correlation between age and occupational self-efficacy (*r* = 0.30, *p* < 0.01). This indicates that there might be a complex interplay between age, perceived workplace age discrimination, and occupational self-efficacy. Certainly, as previously mentioned, the low structural validity of the WADS-G in this particular age group could also be a contributing factor to the overestimation of the correlations.

### 5.3 Internal validity

The findings regarding the weights analysis of the variance explanation in the criterion variables by the WADS-G differ somewhat from those reported in the original study (Marchiondo et al., 2016). Although the WADS-G exhibits a significant association with job satisfaction, turnover intention, occupational self-efficacy, and affective commitment, and explains a meaningful amount of variance in these variables, its predictive power is notably weaker compared to the workplace incivility scale. In relation to the difficulties mentioned above regarding the inability of the scale to distinguish between incivility and ageist discriminatory behavior, these findings of low predictive power support the earlier arguments, as the WADS-G shows an overlap with the incivility scale and might fail to place enough emphasis on ageist discriminatory behavior. Furthermore, the WADS-G showed limited explanatory power for turnover intention when controlling for job satisfaction, work environment, neuroticism, and core self-evaluation. This also raises inquiries regarding the practical relevance of the WADS-G in predicting turnover intention within the employees’ everyday work life. However, it is important to interpret the results in light of the knowledge that the structural validity of the core self-evaluation scale was found to be low (Table 2).

### 5.4 Internal consistency

Considering the existence of specific recommendations and guidelines for evaluating the reliability of unidimensional models, it is imperative to address these aspects, especially given the notable high values of both Cronbach’s alpha and McDonald’s Omega scores. Firstly, the scale’s limited size, comprising only nine items, eliminates the possibility of the high scores being solely influenced by the scale’s length. In this context, Schermelleh-Engel and Gäde (2020) propose that omega coefficients should ideally reach a minimum value of 0.70 or preferably fall within the range of 0.80–0.90 and a narrow confidence interval (CI) should be ensured. Scores exceeding *α* = 0.90 could indicate potential redundancy and content repetition among the included items, as highlighted by Streiner (2003). Marchiondo et al. (2016) reported *α*-scores of 0.93, while Peng et al. (2023) reported even a higher α-score of 0.96. The *α*- and *ω*-scores in our investigation ranged from 0.92 to 0.95 and 0.93 to 0.97, respectively. Notably, the 31–49 sample exhibited the highest *ω*-score, being the only age group where the model demonstrated a moderate measurement fit. Hence, the findings of the current study are consistent with align with prior research and might indicate potential redundancy. This should be especially considered because some items due to their wordings, might address similar issues in their narratives (see Table 1).

### 5.5 Limitations and outlook

In addition to the aforementioned limitations discussed in the previous sections, there are additional limitations that should be taken into account when interpreting the findings of this study. Firstly, participants could only take part in the study via an online survey. Therefore, the study might not have reached a representative sample of individuals and thus lack generalizability. It could be argued that, according to Gosling et al. (2004), online recruitment should not have a major impact on the results but due to the focus of the research, many representative people might not have been reached by the 50+ sample (Bitkom Research, 2020). Secondly, the mean age of the 50+ age group is 55 years, which raises questions about the representativeness of this group in relation to the realistic population of older employees, especially considering that no participants beyond retirement age participated. This is crucial for accurately validating the construct of workplace age discrimination within this specific age group. Especially in light of the increasing number of people who will be working beyond retirement age in the future (Federal Statistical Office of Germany, 2023). Third, despite testing only a unidimensional structure with nine items, previous studies (e.g., Hu and Bentler, 1999) suggest that the sample sizes of certain group-specific samples of this study could ideally have been larger. This limitation particularly effects the age samples 18–30 (*N* = 139) and 50+ (*N* = 168) and might have resulted in a reduced statistical power of the analysis. Fourth, although commonly found in psychological research, it should be noted that not all scales have a normal distribution in the data and the results must be viewed critically based on the statistical methods used (Bishara and Hittner, 2012). And lastly, as mentioned previously, intersectionality might influence measuring the experiences of workplace age discrimination for specific groups. In this context, it is acknowledged that the inclusion of only two genders is not representative for all possible individuals and further research should be conducted in this regard.

In light of the results presented, practitioners should use this scale with caution, given the number of construct-related questions that remain unanswered. However, on a positive note, it also provides ample opportunity for the development of measurement instruments capable of capturing the diverse experiences of age discrimination among various groups of employees. In particular, this opens the door to employing both best practices and innovative approaches from the field of psychometric methods to better understand and address the multifaceted nature of workplace age discrimination. Especially when focusing on researching social interactions, well-crafted questionnaires serve a significant purpose. They not only offer the possibility to make complex phenomena and their impact measurable, but their content can also be used in practice for a better awareness of the experiences of specific groups. Often, only awareness of a complex, subtle phenomenon such as discrimination can enable it to be seen, named and stopped (Becker and Swim, 2011). In addition, items representative of a specific group can help those affected by experiences of discrimination to understand that they are not alone in their experiences and that this is not an individual, but a structural problem.

## Data availability statement

The raw data supporting the conclusions of this article will be made available by the authors, without undue reservation.

## Ethics statement

Ethical approval was not required for the studies involving humans because Ethical review and approval were not required for the study on human participants in accordance with the local legislation and institutional requirements. The studies were conducted in accordance with the local legislation and institutional requirements. The participants provided their written informed consent to participate in this study.

## Author contributions

MF: Conceptualization, Data curation, Formal analysis, Methodology, Writing—original draft. TL: Conceptualization, Methodology, Resources, Supervision, Writing—review & editing.
